# Investigation of Meibomian Gland Function and Dry Eye Disease in Patients with Graves’ Ophthalmopathy †

**DOI:** 10.3390/jcm9092814

**Published:** 2020-08-31

**Authors:** Sachiko Inoue, Motoko Kawashima, Reiko Arita, Ai Kozaki, Kazuo Tsubota

**Affiliations:** 1 Department of Ophthalmology, Keio University School of Medicine, 35 Shinanomachi, Shinjuku-ku, Tokyo 160-8582, Japan; satchin0809@gmail.com (S.I.); ritoh@za2.so-net.ne.jp (R.A.); tsubota@z3.keio.jp (K.T.); 2 Department of Ophthalmology, Itoh Clinic, 626-11 Minaminakano, Minuma-ku, Saitama 337-0042, Japan; 3 Olympia Eye Hospital, 2-18-12 Jingumae Shibuya-ku, Tokyo 150-0001, Japan; ai.koz@i.softbank.jp

**Keywords:** graves’ ophthalmopathy, inflammation, meibomian glands, morphological changes

## Abstract

We prospectively evaluated the relationship between meibomian gland dysfunction (MGD) and Graves’ ophthalmopathy (GO) in 19 patients (38 eyes) with subjective dry eye symptoms, compared to 14 age-matched normal participants (14 eyes). Extraocular muscle and lacrimal gland enlargement were evaluated by magnetic resonance imaging (MRI). Ocular surface examinations included fluorescein staining for keratoconjunctival epithelial damage, tear breakup time (TBUT) evaluation, and Schirmer’s test. Dry eye symptoms were evaluated with the Dry Eye-related Quality-of-Life Score (DEQS) questionnaire. Lid-margin abnormalities, meibum grade, and meiboscores were assessed using meibography. Clinical activity scores and T2 signal intensity ratios were used to define GO activity. All GO patients had obstructive MGD and 79% exhibited levator muscle enlargement. Ocular surface parameters of TBUT (*p* = 0.000), meibum score (*p* = 0.000), eyelid vasculitis (*p* = 0.000), meiboscore of the upper lid (*p* = 0.002), total meiboscores (*p* = 0.001), and DEQS (*p* = 0.000) significantly differed between GO patients and normal subjects. In addition, GO patients had significantly more abnormalities of the central region of the upper eyelid than normal subjects (*p* = 0.000). Thus, MGD might be related to eye discomfort and deterioration of the ocular surface in GO patients. Inflammation and morphological meibomian gland changes might be characteristic of GO.

## 1. Introduction

Graves’ ophthalmopathy (GO) is a periocular and orbital inflammatory manifestation that is caused by autoimmune thyroid disease [1]. The pathogenesis of GO involves autoantibodies against thyroid-stimulating hormone (TSH) receptors, which lead to excess production of thyroid hormone. This, in turn, induces an inflammatory response against the periocular and orbital tissues [2,3,4]. Dry eye disease (DED) is a frequent complaint among patients with GO [2,5], and a high proportion of patients with GO reportedly exhibit dry eye symptoms [5,6]. However, although numerous studies have investigated the pathogenesis of GO, few have offered detailed insight into the association and interaction between GO and DED [2,3,4,5,6,7,8,9]. Moreover, in clinical settings, DED in GO tends to remain neglected.

Regarding the DED mechanism in GO, previous studies have suggested (1) a decrease in tear volume, because the lacrimal gland is one of the target tissues of TSH [2,3,4], and (2) an increase in palpebral fissure height, leading to accelerated evaporation of tears [10]. Recent studies have suggested that GO pathogenesis is mediated not only by morphological changes but also by immunological causes, including T-cell-mediated inflammation [3] and TSH-receptor expression in the acinar cells of impaired lacrimal glands [2].

Meibomian glands secrete the lipid component of tears, which prevents excessive tear evaporation. Meibomian gland dysfunction (MGD) is considered to be a major cause of evaporative dry eye [11,12,13]. However, few reports have investigated the relationship between GO and the ocular surface, including both MGD and DED. Therefore, here we investigated the relationship between GO and MGD in patients with GO and dry eye symptoms, using detailed GO information obtained using various modalities, such as magnetic resonance imaging (MRI).

## 2. Experimental Section

### 2.1. Patients

This study was a prospective, clinic-based, descriptive study and adhered to the guidelines of the Declaration of Helsinki (as amended in 2013). The study protocol was approved by the institutional review board of the Keio University School of Medicine (No. 20120210). All patients received a full explanation of the study procedures and written informed consent was obtained from each subject prior to enrolment. To ensure privacy, all records were identified by an anonymous subject identification number.

All GO patients were referred to our clinic from Olympia Eye Hospital, Tokyo, Japan. They were diagnosed with GO in accordance with previously described criteria [14]. Additionally, the patients underwent diagnostic imaging by MRI at the hospital. The patients with subjective dry eye symptoms—such as dry eye sensation, foreign body sensation, and photophobia—had been referred to the Keio University Hospital MGD clinic consecutively. Normal participants without GO were also included in this study as controls. All patients underwent a general ophthalmic examination and slit-lamp biomicroscopy. Furthermore, they underwent a more detailed examination of MGD and the ocular surface. 

### 2.2. Ocular Surface Examination

Two investigators with experience of investigation of the ocular surface (M.K. and S.I.) evaluated the tear breakup time (TBUT) as well as keratoconjunctival epithelial damage, on the basis of fluorescein staining scores (0–9) of the cornea and conjunctiva, as described previously [15]. For these tests, 2 μL of preservative-free 0.5% fluorescein dye was instilled into each eye using a micropipette, to avoid altering the tear dynamics. Then, conjunctival rose bengal staining was performed to detect superior limbic keratoconjunctivitis (SLK) and lid wiper syndrome. Finally, Schirmer’s test was performed without topical anesthesia. 

### 2.3. Criteria for Dry Eye Diagnosis

A dry eye diagnosis was made according to the latest Japanese dry eye diagnostic criteria (2016) [16]. Briefly, the presence of dry eye symptoms and the presence of qualitative disturbance of the tear film (TBUT ≤ 5 s) both had to be present for a diagnosis of dry eye [16]. 

### 2.4. Criteria for Diagnosis of Meibomian Gland Dysfunction

Obstructive MGD was diagnosed when an eye tested positive for all 3 of the following criteria: (1) symptoms of MGD, such as dry eye sensation, burning sensation, foreign body sensation; (2) abnormal findings around the orifices of the glands, and (3) findings indicating meibomian gland orifice obstruction. The presence of any two or more of these findings was defined as a lid margin abnormality in this study. An eye was judged positive for abnormal findings around the orifices when at least 1 of 3 findings, i.e., irregular lid margin, vascular engorgement, or anterior/posterior displacement of the mucocutaneous junction, was recognized. Vascular engorgement was defined as the presence of moderate or severe telangiectasia or redness. An eye was judged positive for orifice obstruction when both findings indicative of meibomian gland orifice obstruction, i.e., decreased meibomian secretion, and plugging, pouting, and ridging were recognized [17]. Meibomian secretion (meibum) was graded as follows: grade 0, clear meibum, easily expressed; grade 1, cloudy meibum, expressed with mild pressure; grade 2, cloudy meibum, expressed with more than moderate pressure; and grade 3, no meibum expression, even with hard pressure [18]. Morphological changes in the meibomian glands were observed using a non-contact mobile meibography system (Japan Focus Corporation, Tokyo, Japan) and graded accordingly [19]. Partial or complete meibomian gland loss was scored using the following grades for each eyelid, as previously described: grade 0, no meibomian gland loss; grade 1, area of meibomian gland loss < 1/3 of the total meibomian gland area; grade 2, area of meibomian gland loss between 1/3 and 2/3 of the total area; and grade 3, area of meibomian gland loss > 2/3 of the total area. Meiboscores (0–6) were summed to obtain a score from 0 through 6 for each eye [20]. The central region was also defined as the middle 1/3 part of the lid width.

### 2.5. Examination for Graves’ Ophthalmopathy

Proptosis (protrusion > 15 mm) was measured using a Hertel exophthalmometer (Handaya, Tokyo, Japan). Lid retraction was defined as a palpebral fissure height > 7 mm [14] and exposure of the upper sclera. In the MRI investigation, levator muscle enlargement was determined in a sagittal section. Swelling of the lacrimal gland and extraocular muscle enlargement was determined in a coronal section. Muscles that were clearly thicker than the optic nerve were determined to be enlarged. GO activity was defined based on clinical activity score (CAS) [21] and T2 signal intensity ratios (T2SIR) [22]. Each patient was assigned a CAS after examination by an ophthalmologist. This score is based on 4 well-known classic signs of inflammation, i.e., pain, redness, swelling, and impaired function, and consists of scores for 10 items. Each sign judged as present is scored 1 point, and each sign has the same weight. The active phase of GO was defined by CAS ≥ 4 points. The T2SIR was defined by the ratio of signal strengths of extraocular muscles and the ipsilateral temporal muscle on T2-weighted images. 

The severity of GO was classified using Olympia Eye Hospital diagnostic criteria. The classification of mild GO was as follows: palpebral fissure height was 7–10 mm, lid swelling was mild (eyelids mildly swollen with fluid), the conjunctiva showed chemosis, injection, or congestion; the range of proptosis was 15–18 mm, the extra-ocular muscles exhibited no or intermittent diplopia. Additionally, no optic nerve, retina, or corneal findings were exhibited.

The classification of moderate GO was as follows: palpebral fissure height was 10–12 mm; lid swelling was moderate (eyelid skin showed obvious swelling but the tissue was not tense); the conjunctiva showed SLK; the range of proptosis was 18–21 mm; the extra-ocular muscle showed disorder, as exhibited by diplopia of the peripheral field-of-view; and the cornea showed infiltration due to lagophthalmos that affected the entire cornea. Additionally, no optic nerve or retina findings were exhibited.

Severe GO was classified as severe if the following were present: palpebral fissure height was 12 mm or more, lid swelling was severe (the upper eyelid skin-fold was ballooned out, filled with fluid, and the skin was taught), the conjunctiva showed upper scleral vessel engorgement, the range of proptosis was 21 mm or more, the extra-ocular muscle disorder extended to diplopia of the 1st eye position, the cornea shows infiltration due to lagophthalmos, affecting the entire cornea. Additionally, no optic nerve or retina findings were present. 

Furthermore, the most severe form of GO was classified when, in addition to the findings of severe GO, corneal perforation, ulcer, or optic neuropathy were present. If even one of these findings was present, the condition was regarded as the most severe GO.

### 2.6. Assessment of Subjective Symptoms, Self-Reported via a Questionnaire

A validated dry eye symptom questionnaire, the Dry Eye-related Quality-of-Life Score (DEQS) questionnaire, was administered. The DEQS questionnaire was recently developed in Japan and its internal consistency, test-retest reliability, discriminant validity, and responsiveness to change have all been validated previously [23]. It comprises 15 questions; 6 questions assess ocular symptoms and 9 assess the effect of DED on the quality of life. The 6 questions related to ocular symptoms query respondents on the presence and severity of foreign body sensations, dry eye sensations, pain or soreness, ocular fatigue, eyelid heaviness, and eye redness. The frequency of symptoms is scored from 0 (none) to 4 (highest frequency), and the severity of symptoms is scored from 1 (low) to 4 (high) [23]. The summary scale score ranges from 0 (best) to 100 (worst).

### 2.7. Statistical Analysis 

Data were analyzed using the Statistical Package for the Social Sciences version 26.0 (IBM Corp., Armonk, NY, USA) and Excel^®^ version 14.1.0 (Microsoft^®^, Redmond, WA, USA). The t-test was used for continuous variables, the Mann–Whitney U-test for ordinal variables and chi-squared test for nominal variables. Statistical significance was indicated at *p* < 0.05.

## 3. Results

In total, 36 patients were included in this study; 19 were patients with GO and 14 were normal participants. Table 1 shows the characteristics of the 19 GO patients, among whom 1 (2 eyes) had active GO, 18 had hyperthyroidism, and 1 had hypothyroidism. The mean duration of GO was 52.9 ± 21.5 months (Table 1). The mean age of all patients was 44.1 ± 7.9 years (range, 27–56 years). The mean age of GO patients was 44.0 ± 10.0 years (range, 27–56 years) and that of control participants was 44.6 ± 7.6 years (range, 27–56 years). There were no statistically significant differences between GO patients and normal patients (*p* = 0.4). The GO patients comprised of 17 women and 2 men whereas normal participants were all women (Table 2).

### 3.1. Ocular Surface Examination

**Figure 1:**
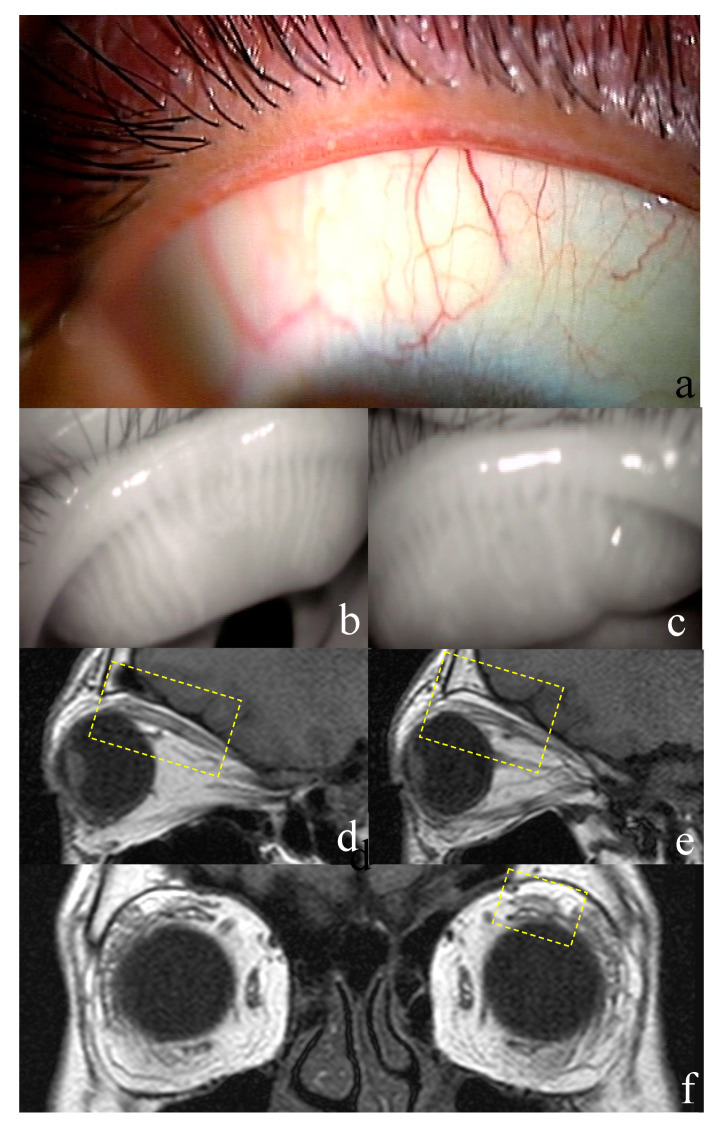
Representative case of a 53-year-old woman with Graves’ ophthalmopathy. The patient presented with vascular engorgement, lid-margin inflammation, and plugging of the lid margin (**a**). The meibomian glands at the central region of the eyelids exhibit abnormal findings, particularly in the upper eyelid (**b**,**c**). Magnetic resonance imaging findings show enlargement of the levator muscle and lacrimal gland (**d**–**f**).

**Figure 2:**
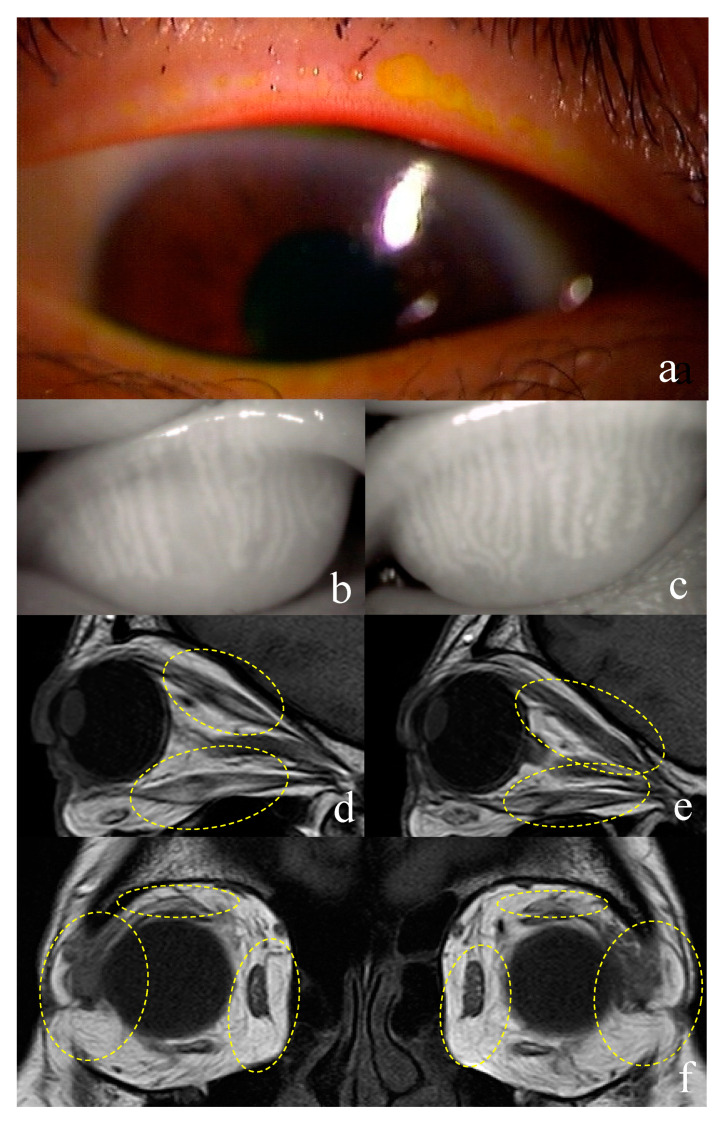
Representative case of a 56-year-old woman with Graves’ ophthalmopathy. The patient presented with lid-margin inflammation (**a**). The meibomian glands in the central region of the upper eyelid exhibit abnormal findings (**b**,**c**). Magnetic resonance imaging findings show (**d**,**e**) levator muscle and superior and inferior rectus muscle enlargement, as well as (**f**) medial rectus muscle enlargement and swelling of the lacrimal glands.

In ocular surface investigations, the right eye was selected in GO patients, and their 19 eyes were compared to the 14 eyes of normal participants. Overall, 32 of 38 eyes (84.2%) of patients with GO were diagnosed with DED. Notably, all patients exhibited obstructive MGD. Objective ocular surface parameters are summarized in Table 2. TBUT was significantly shorter in patients with GO than that in normal participants (*p* = 0.000; Table 2). Vasculitis, meibum score, and DEQS were significantly higher in patients with GO than those in normal participants (*p* = 0.000, *p* = 0.000, *p* = 0.000 respectively; Table 2). Upper eyelid meiboscore and total meiboscore (upper and lower eyelid) were significantly higher in patients with GO than in normal participants (*p* = 0.002, *p* = 0.001 respectively; Table 2). Furthermore, abnormalities of the central part of the upper eyelid were significantly more common in patients with GO than in normal participants (*p* = 0.000; Table 2). The fluorescein staining score, Schirmer’s test findings, and meiboscore of the lower lid were not significantly different between the two groups. There were no patients with SLK (Table 2; Figure 1 and Figure 2).

### 3.2. Examination for Graves’ Ophthalmopathy 

Proptosis was observed in 38 of 38 eyes (100%); the mean proptosis value was 18.8 ± 1.8 mm. The mean palpebral fissure height was 9.0 ± 2.0 mm; of the 38 eyes, 33 (91.7%) exhibited palpebral fissure height > 7 mm. Twenty of 38 eyes (52.6%) exhibited thickening of the eyelids (eyelid swelling). Engorgement of the levator muscle on MR images was seen in 30 of 38 eyes (78.9%) (Figure 1; Figure 2). Engorgement of the extra-ocular muscles on MR images was seen in 17 of 38 eyes (44.7%). Lacrimal gland swelling was observed in 28 of 38 eyes (73.7%), and eyelid retraction was observed in 4 of 38 (10.5%) eyelids (Table 1). In terms of severity of GO, 10 patients were classified as having mild and 9 patients were classified as having moderate disease (Table 1). Only 1 patient was in the acute phase (Table 1).

Detailed data of the ocular surface parameters and GO parameters of the patients are presented in Table 3.

### 3.3. Representative Cases

#### 3.3.1. Case 1

A 53-year-old woman presented with GO, (disease duration of 60 months), in the inactive phase at presentation. The DEQS score was 25. The patient exhibited eyelid swelling, as well as exophthalmos (19 mm and 18 mm in the right and left eyes, respectively; Figure 1). There was no obvious eyelid retraction; the palpebral fissure height of the right and left eyes was relatively normal and mild (7 mm and 8 mm, respectively). The patient complained of dry eye symptoms, and the TBUT in both eyes was 6 s. In both eyes, the corneo-conjunctival staining score was 1, and the Schirmer’s test value was 2 mm. The eyelid margins exhibited plugging (upper lid margin), displacement of the mucocutaneous junction, vascular engorgement (upper and lower lid margins), and inflammation (eyelid and ocular conjunctiva) (Figure 1d). The meibum grade was 1. On the basis of these findings, the patient was diagnosed with MGD. Meibography findings indicated meibomian gland dropout in the upper and lower eyelids of the right eye and the central region of the upper eyelid of the left eye (Figure 1b,c,e,f). Levator muscle swelling in the upper eyelids and lacrimal gland swelling were observed in MR images (Figure 1g–i). The CAS score was 0.

#### 3.3.2. Case 2

A 56-year-old woman presented with GO (disease duration of 96 months), in the inactive phase at presentation. The DEQS score was 8.3. Initially, the patient exhibited swelling, chronic inflammation, and pigmentation of the eyelids. Exophthalmos was observed in both eyes (17 mm and 16 mm in the right and left eyes, respectively; Figure 2a). In the follow-up evaluation (current status), no lid retraction was observed, and the palpebral fissure height of both eyes was mild (8 mm, both eyes). The patient complained of dry eye symptoms at presentation, and the TBUT in the right and left eyes was 3 s and 5 s, respectively. The corneo-conjunctival score in both eyes was 0. Schirmer’s test values in the right and left eyes were 4 mm and 13 mm, respectively. Evaluation of eyelid margins revealed plugging and vascular engorgement (upper and lower eyelid margins) as well as inflammation (palpebral and ocular conjunctiva) (Figure 2d). Meibum grades in the right and left eyes were 2 and 1, respectively. On the basis of these findings, the patient was diagnosed with DED and MGD. Meibography findings revealed meibomian gland dropout in the upper and lower eyelids of both eyes; both eyes exhibited a meiboscore of 3 (Figure 2b,c,e,f). Levator muscle swelling in the upper eyelid, as well as swelling of the superior, medial, and inferior rectus muscles and lacrimal glands, were observed on MR images (Figure 1g–i). The CAS score was 1.

## 4. Discussion

This study investigated the relationship between ocular surface parameters, including MGD and DED, and GO in patients with dry eye symptoms. A high percentage (84.2%) of the patients were diagnosed with DED. More surprisingly, all patients in this study exhibited obstructive MGD; this proportion was higher than the proportion of patients with DED. Our findings revealed that patients presented with clinical features of vascular engorgement and inflammation of eyelid margins, levator muscle thickening, and in some patients, meibomian gland changes in the central region of the eyelids, based on meibography. TBUT, vasculitis, DEQS, and meiboscores (upper lid and total score) were significantly worse in GO patients than those in the normal controls. Recently, Kim et al. reported that patients with GO exhibited morphological changes in the meibomian glands, which correlated with proptosis and palpebral fissure height [24]. The authors considered that lack of blinking by proptosis and palpebral fissure height in patients with GO was caused by decreased excretion of meibum and gave rise to obstructive MGD. The patients in this study had high meibum grades, which were similar to those previously reported in 60-year-old patients with MGD [25,26]. Recent studies reported that oxidative stress is associated with GO [27,28]. According to another study, oxidative stress resulted in changes in the meibomian glands and meibum composition [29]. In this study, most patients exhibited proptosis and increased palpebral fissure height. Based on previous studies and the results of the present study, we speculated that MGD and deterioration of meibum were caused by morphological changes related to GO and oxidative stress. The meiboscores of patients with GO were significantly higher than that of normal participants in this study, and those previously reported [24]. Furthermore, we found that patients with GO exhibited meibomian gland changes in the central region of the eyelids, particularly of the upper lid, which has not been reported previously. In a previous study of contact-lens users [30], meibography findings revealed negative changes. Hence, the authors concluded that this condition was correlated to the duration of use and chronic irritation caused by the contact-lenses. Most patients (nearly 80%) in the study exhibited levator muscle enlargement. This position might correspond to the superior levator muscles and also SLK. SLK is often observed in patients with GO and is caused by abnormal friction and ocular surface inflammation [31,32]. In a previous study, SLK had a stronger correlation with lid retraction and extra-ocular muscle enlargement in GO patients than in individuals without GO [31,33]. Although, none of our patients exhibited SLK and eyelid retraction, because most patients were in the inactive phase of GO. These results suggest that levator muscle condition may affect the upper central meibomian gland morphology. However, considering the results of our own and previous reports, patients with meibography changes in the center of the eyelids might have inflammation and friction. Furthermore, they may have SLK and lid retraction during the active phase of GO. Hence, we speculated that friction and inflammation may be caused by GO; prolonged duration of GO might lead to changes in the center of the eyelids that are noted by meibography. We also hypothesized that these findings would be a characteristic meibography sign in some GO patients with dry eye symptoms. As in previous studies, TBUT in this study was lower than normal participants. [3,7,8,24]. Proptosis and increased palpebral fissure height have been reported as causes of ocular surface damage in patients with GO [2,4]. In particular, increased palpebral fissure height has been reported to be the most influential factor for tear evaporation [10,34,35]. In our study, most patients exhibited proptosis and increased palpebral fissure height; therefore, we speculated that these anatomical factors were among the reasons for the relatively short TBUT. Furthermore, excessive tear evaporation has been proposed as a cause of hyperosmolarity [35,36].

Tear hyperosmolarity stimulates the production of proinflammatory cytokines at the ocular surface [35,36]. These inflammatory responses are known to be related to T-lymphocytes. It has been reported that GO is caused by T-lymphocyte-mediated stimulation of orbital cells [2,4]. Expression of TSH receptors in GO patients has also been demonstrated, and the lacrimal gland is known as the target organ for TSH [2]. Previously, computed tomography findings have revealed swollen lacrimal glands in patients with GO [37]. In this study, MRI findings showed lacrimal gland swelling, and our patients’ Schirmer’s test values were lower than those reported in previous studies [7,33]. The rate of vascular engorgement of the lid margin in this study was 97.3%, although, a previous study showed that the rate of this phenomenon in normal controls in women of the same age range was only 14% [20]. We also recognized inflammatory signs around the eyes. Since GO is caused by inflammation and immune-related mechanisms, GO is likely to result in MGD and DED.

This study had a few limitations. First, this was a pilot study and, therefore, the sample size was small; however, we will be conducting a similar study with a larger sample size in the future and include additional comparisons such as those of the active and inactive phases of GO. Second, some selection bias might have occurred, although we included consecutive patients with GO. Inclusion of patients with GO at different stages of activity (active/chronic) and different disease durations might have helped to define the details of the relationship between GO and MGD more specifically, thereby helping to elucidate the point of occurrence of MGD in GO eyes. In addition, in further studies, quantitative markers, such as those reflecting ocular surface oxidative stress and inflammation, should be studied in patients with GO. Furthermore, we would like to investigate the pathophysiological mechanisms and inflammatory pathways (e.g., T-lymphocyte-related pathways and oxidative stress-related pathways) related to MGD in GO patients, using experimental models. 

## 5. Conclusions

In summary, we found strong associations between dry eye symptoms in GO patients and ocular surface parameters, including MGD and DED. These were characterized by meibomian gland dropout at the center of the eyelids, as well as eyelid margin vascular engorgement and inflammation. Inflammation and morphological changes of meibomian glands might be characteristic findings in patients with GO. Although further investigations are required to elucidate the mechanism underlying the association between the ocular surface state, including MGD and DED, and GO, the present findings indicate the possibility that MGD may be involved in causing eye discomfort in patients with GO.

## Figures and Tables

**Table 1:** Clinical characteristics and ophthalmologic parameter of the 19 patients with Graves’ ophthalmopathy.

Parameter	Patients with GO (*n* = 19)
Clinical parameters	
Duration of GO, months	52.9 ± 21.5
Active GO, *n* (CAS ≥ 4)	1
Smokers, *n* (%)	8 (42.1)
Thyroid function	
Hyperthyroidism, *n*	18
Hypothyroidism, *n*	1
Euthyroidism, *n*	0
Ophthalmologic parameter	
Severity of GO, mild *n*, (%)	10/19 (52.6)
Moderate *n* (%)	9/19 (47.4)
Proptosis (>15 mm) *n*, mm, (%)	38/38, 18.8 ± 1.8, (100)
Eyelid retraction, *n* (%)	4/38 (10.5)
Palpebral fissure height (>7 mm) *n*, mm, (%)	33/38, 9.0 ± 2.0 (91.7)
Eyelid swelling, *n* (%)	22/38 (57.9)
Engorgement of the levator muscle, *n* (%)	30/38 (78.9)
Engorgement of the extra ocular muscle, *n* (%)	17/38 (44.7)
Swelling of the lacrimal gland, *n* (%)	28/38 (73.7)

GO: Graves’ ophthalmopathy, CAS: clinical activity score.

**Table 2:** Clinical parameters of ophthalmic evaluations.

Parameter	Patients with GO (*n* = 19)	Normal Participants (*n* = 14)	*p* Value
Mean age, years (mean ± SD)	44.0 ± 10.0	44.6 ± 2.2	*p* = 0.6
Male, *n*	2	0	*p* = 0.4
Ocular surface examination			
Lid margin abnormalities, n	19		
Vasculitis, *n*	19	2	*p* = **0.000**
Fluorescein score	0.6 ± 0.8	0.3± 0.5	*p* = 0.4
TBUT, s	4.7 ± 3.2	7.1 ± 1.6	*p* = **0.000**
SLK, *n*	0	0	-
Schirmer’s test, mm	10.5 ± 8.8	9.2 ± 3.5	*p* = 0.5
Meibum score	1.6 ± 0.5	0.3 ± 0.5	*p* = **0.000**
Meibography			
Meiboscores			
Upper lid	1.0 ± 0.6	0.3 ± 0.5	*p* = **0.002**
Lower lid	0.9 ± 0.6	0.4 ± 0.5	*p* = 0.07
Total meiboscores	1.9 ± 1.1	0.7 ± 0.8	*p* = **0.001**
Abnormalities of central part, *n*	12	0	*p* = **0.000**
DEQS	35.2 ± 28.3	4.3 ± 1.3	*p* = **0.000**

TBUT = tear breakup time; SLK = superior limbic keratoconjunctivitis; DEQS = Dry Eye-related Quality-of-Life Score, GO: Graves’ ophthalmopathy. All values are expressed as mean ± standard deviation (SD). *p*-value, vs. normal participants.

**Table 3:** Ocular surface parameters and Graves’ ophthalmopathy (GO) severity.

										Meibography											Engorgement		Swelling		
				Fluorescein		Lid margin Vasculitis		Schirmer‘s Test (mm)		Meiboscore			Abnormalities Central Part												
	Age	Sex	Eye	Score	TBUT (s)	Upper Lid	Lower Lid		Meibum Grade	Upper Lid	Lower Lid	Total	Upper Lid	Lower Lid	DEQS	CAS	GO Severity	Disease Duration	Proptosis	Palpebral Fissure	Levator Muscle	Extra-Ocular Muscles	Lacrimal Gland	Eyelid Swelling	Lid Retraction
1	53	F	R	1	6	1	1	2	1	2	2	4	1	0	25	0	2	60	19	7	1	0	1	1	0
			L	1	6	1	1	2	1	2	0	2	1	0					18	8	1	0	1	1	0
2	56	F	R	2	6	1	1	23	2	1	0	1	1	0	55	5	2	53	19	8	1	0	1	1	0
			L	3	4	1	1	13	2	1	0	1	0	0					18	10	1	1	1	1	0
3	41	F	R	0	4	0	1	9	2	0	0	0	0	0	60	2	1	58	18	8	1	0	1	0	0
			L	0	4	1	1	9	2	0	0	0	0	0					17	9	1	1	1	0	0
4	38	F	R	0	4	1	1	6	1	2	1	3	0	0	55	0	2	60	20	8	1	0	1	1	0
			L	0	3	1	1	4	1	2	1	3	0	0					21	9	1	0	1	1	0
5	47	F	R	1	2	1	1	0	2	0	1	1	1	1	56.7	0	1	24	16	10				0	0
			L	1	2	1	1	4	2	0	1	1	1	0					16	9				0	0
6	38	F	R	0	3	1	1	19	1	1	1	2	0	0	11.6	2	2	60	19	8	1	0	1	1	0
			L	0	4	1	1	17	1	1	0	1	0	0					22	12	1	0	1	1	0
7	42	F	R	0	2	1	1	2	1	1	1	2	1	0	16.6	0	1	24	19	8	0	0	0	0	0
			L	0	2	1	1	0	1	1	2	3	1	0					18	10	0	0	0	0	0
8	28	F	R	1	0	1	1	3	1	1	0	1	1	1	26.7	2	2	48	20	11	1	1	1	1	1
			L	1	0	1	0	2	1	1	0	1	1	1					19	11	1	1	1	1	1
9	48	F	R	0	4	1	1	0	2	2	1	3	1	0	23.3	0	1	60	18	10	1	0	1	0	0
			L	0	4	1	1	1	2	1	1	2	1	0					18	9	1	0	1	0	0
10	39	F	R	2	8	1	1	11	2	1	1	2	0	0	30	1	2	7	19	10	1	1	0	1	0
			L	2	5	1	1	12	2	1	1	2	0	0					19	11	1	1	0	1	0
11	44	F	R	1	4	1	1	6	2	1	1	2	1	0	20	1	1	65	18	6	0	0	0	0	0
			L	1	3	1	1	11	2	1	1	2	1	0					19	8	0	0	0	0	0
12	39	M	R	0	5	1	1	17	1	1	0	1	1	1	21.7	1	1	75	18	9	1	0	1	0	0
			L	0	5	1	1	11	1	1	0	1	1	0					18	9	1	0	1	0	0
13	67	F	R	0	5	1	1	14	2	2	1	3	1	0	35.7	0	1	72	18	8	1	1	1	0	0
			L	0	5	1	1	12	2	1	1	2	0	0					17	8	1	1	1	0	0
14	29	F	R	0	5	1	1	20	1	0	0	0	1	0	3.3	1	1	24	18	7	1	0	1	1	0
			L	0	3	1	1	14	1	0	0	0	0	0					19	9	1	1	1	1	0
15	48	M	R	0	3	1	1	14	2	1	1	2	1	0	58.9	2	2	64	23	9	1	1	1	0	0
			L	0	3	1	1	15	2	1	1	2	0	0					21	9	1	1	1	0	0
16	27	F	R	1	5	1	1	12	2	1	3	4	0	0	10	1	2	48	22	12	1	1	1	1	0
			L	0		1	1	22	2	1	3	4	0	0					23	12	1	1	1	1	0
17	56	F	R	1	3	1	1	3	2	1	3	4	0	0	8.3	1	2	55	18	8	1	1	1	1	0
			L	1	2	1	1	3	2	1	2	3	0	0					20	8	1	1	1	1	1
18	38	F	R	0	6	1	1	35	1	1	1	2	0	0	91.7	1	1	8	16	6	1	1	1	1	1
			L	0	7	1	1	35	1	1	1	2	1	0					16	7	1	1	1	1	0
19	52	F	R	0	3	1	1	4	2	2	1	3	1	0	58.9	0	1	96	17	8	1	0	0	1	0
			L	0	5	1	1	13	1	2	1	3	1	0					16	8	1	0	0	1	0

TBUT: tear breakup time, DEQS: Dry eye-Related Quality-of-Life Score, F: female, M: male, R: right, L: left.

## References

[1] Bothun E.D., Scheurer R.A., Harrison A.R., Lee M.S. (2009). Update on thyroid eye disease and management. Clin. Ophthalmol..

[2] Bahn R.S., Dutton C.M., Natt N., Joba W., Spitzweg C., Heufelder A.E. (1998). Thyrotropin receptor expression in Graves’ orbital adipose/connective tissues: Potential autoantigen in Graves’ ophthalmopathy. J. Clin. Endocrinol. Metab..

[3] Lehmann G.M., Feldon S.E., Smith T.J., Phipps R.P. (2008). Immune mechanisms in thyroid eye disease. Thyroid.

[4] Eckstein A.K., Finkenrath A., Heiligenhaus A., Renzing-Köhler K., Esser J., Krüger C., Quadbeck B., Steuhl K.P., Gieseler R.K. (2004). Dry eye syndrome in thyroid-associated ophthalmopathy: Lacrimal expression of TSH receptor suggests involvement of TSHR-specific autoantibodies. Acta Ophthalmol. Scand..

[5] Gürdal C., Saraç O., Genç I., Kırımlıoğlu H., Takmaz T., Can I. (2011). Ocular surface and dry eye in Graves’ disease. Curr. Eye Res..

[6] Coulter I., Frewin S., Krassas G.E., Perros P. (2007). Psychological implications of Graves’ orbitopathy. Eur. J. Endocrinol..

[7] Selter J.H., Gire A.I., Sikder S. (2014). The relationship between Graves’ ophthalmopathy and dry eye syndrome. Clin. Ophthalmol..

[8] Villani E., Viola F., Sala R., Salvi M., Mapelli C., Currò N., Vannucchi G., Beck-Peccoz P., Ratiglia R. (2010). Corneal involvement in Graves’ orbitopathy: An in vivo confocal study. Investig. Ophthalmol. Vis. Sci..

[9] Rocha E.M., Mantelli F., Nominato L.F., Bonini S. (2013). Hormones and dry eye syndrome: An update on what we do and don’t know. Curr. Opin. Ophthalmol..

[10] Brasil M.V., Brasil O.F., Vieira R.P., Vaisman M., do Amaral Filho O.M. (2005). Tear film analysis and its relation with palpebral fissure height and exophthalmos in Graves’ ophthalmopathy. Arq. Bras. Oftalmol..

[11] Lemp M.A. (1995). Report of the National Eye Institute/Industry workshop on clinical trials in dry eyes. Clao. J..

[12] Mathers W.D. (1993). Ocular evaporation in meibomian gland dysfunction and dry eye. Ophthalmology.

[13] Shimazaki J., Sakata M., Tsubota K. (1995). Ocular surface changes and discomfort in patients with meibomian gland dysfunction. Arch. Ophthalmol..

[14] Kozaki A., Inoue R., Komoto N., Maeda T., Inoue Y., Inoue T., Ayaki M. (2010). Proptosis in dysthyroid ophthalmopathy: A case series of 10,931 Japanese cases. Optom. Vis. Sci..

[15] Uchino Y., Uchino M., Dogru M., Ward S., Yokoi N., Tsubota K. (2014). Changes in dry eye diagnostic status following implementation of revised Japanese dry eye diagnostic criteria. Jpn. J. Ophthalmol..

[16] Tsubota K., Yokoi N., Shimazaki J., Watanabe H., Dogru M., Yamada M., Kinoshita S., Kim H.M., Tchah H.W., Hyon J.Y. (2017). Asia Dry Eye Society. New perspectives on dry-eye definition and diagnosis: A consensus report by the Asia Dry eye Society. Ocul. Surf..

[17] Meibomian Gland Dysfunction Working Group (2010). Definition and diagnostic criteria for meibomian gland dysfunction. Atarashii Ganka..

[18] Shimazaki J., Goto E., Ono M., Shimmura S., Tsubota K. (1998). Meibomian gland dysfunction in patients with Sjögren syndrome. Ophthalmology.

[19] Arita R., Itoh K., Maeda S., Maeda K., Amano S. (2013). A newly developed noninvasive and mobile pen-shaped meibography system. Cornea.

[20] Arita R., Itoh K., Inoue K., Amano S. (2008). Noncontact infrared meibography to document age-related changes of the meibomian glands in a normal population. Ophthalmology.

[21] Mourits M.P., Prummel M.F., Wiersinga M., Koornneef L. (1997). Clinical activity score as a guide in the management of patients with Graves’ ophthalmopathy. Clin. Endocrinol..

[22] Tortora F., Cirillo M., Ferrara M., Belfiore M.P., Carella C., Caranci F., Cirillo S. (2013). Disease activity in Graves’ ophthalmopathy: Diagnosis with orbital MR imaging and correlation with clinical score. Neuroradiol. J..

[23] Sakane Y., Yamaguchi M., Yokoi N., Uchino M., Dogru M., Oishi T., Ohashi Y., Ohashi Y. (2013). Development and validation of the Dry-eye-Related Quality-of-Life Score questionnaire. JAMA Ophthalmol..

[24] Kim Y.S., Kwak A.Y., Lee S.Y., Yoon J.S., Jang S.Y. (2015). Meibomian gland dysfunction in Graves’ orbitopathy. Can. J. Ophthalmol..

[25] Arita R., Morishige N., Koh S., Shirakawa R., Kawashima M., Sakimoto T., Suzuki T., Tsubota K. (2015). Increased tear fluid production as a compensatory response to meibomian gland loss: A multicenter cross-sectional study. Ophthalmology.

[26] Arita R., Minoura I., Morishige N., Shirakawa R., Fukuoka S., Asai K., Goto T., Imanaka T., Nakamura M. (2016). Development of definitive and reliable grading scales for meibomian gland dysfunction. Am. J. Ophthalmol..

[27] Tsai C.C., Wu S.B., Cheng C.Y., Kao S.C., Kau H.C., Lee S.M., Wei Y.H. (2011). Increased response to oxidative stress challenge in Graves’ ophthalmopathy orbital fibroblasts. Mol. Vis..

[28] Zarković M. (2012). The role of oxidative stress on the pathogenesis of graves’ disease. J. Thyroid Res..

[29] Ibrahim O.M., Dogru M., Matsumoto Y., Igarashi A., Kojima T., Wakamatsu T.H., Inaba T., Shimizu T., Shimazaki J., Tsubota K. (2014). Oxidative stress induced age dependent meibomian gland dysfunction in Cu, Zn-superoxide dismutase-1 (Sod1) knockout mice. PLoS ONE.

[30] Arita R., Itoh K., Inoue K., Kuchiba A., Yamaguchi T., Amano S. (2009). Contact lens wear is associated with decrease of meibomian glands. Ophthalmology.

[31] Kabat A.G. (1998). Lacrimal occlusion therapy for the treatment of superior limbic keratoconjunctivitis. Optom. Vis. Sci..

[32] Takahashi Y., Ichinose A., Kakizaki H. (2014). Topical rebamipide treatment for superior limbic keratoconjunctivitis in patients with thyroid eye disease. Am. J. Ophthalmol..

[33] Murakami Y., Kanamoto T., Tuboi T., Maeda T., Inoue Y. (2001). Evaluation of extraocular muscle enlargement in dysthyroid ophthalmopathy. Jpn. J. Ophthalmol..

[34] Iskeleli G., Karakoc Y., Abdula A. (2008). Tear film osmolarity in patients with thyroid ophthalmopathy. Jpn. J. Ophthalmol..

[35] Gilbard J.P., Farris R.L. (1983). Ocular surface drying and tear film osmolarity in thyroid eye disease. Acta Ophthalmol..

[36] Achtsidis V., Tentolouris N., Theodoropoulou S., Panagiotidis D., Vaikoussis E., Saldana M., Gouws P., Theodossiadis P.G. (2013). Dry eye in Graves’ ophthalmopathy: Correlation with corneal hypoesthesia. Eur. J. Ophthalmol..

[37] Harris M.A., Realini T., Hogg J.P., Sivak-Callcott J.A. (2012). CT dimensions of the lacrimal gland in Graves orbitopathy. Ophthal. Plast. Reconstr. Surg..

